# Characterization of the bacterial and fungal diversity in habitats of Corsica Island

**DOI:** 10.1128/aem.00756-25

**Published:** 2025-10-01

**Authors:** Sonia Aghzaf, Jean-Pierre Poli, Marion Brunel, Jean Costa, Vannina Lorenzi, Elodie Guinoiseau, Liliane Berti

**Affiliations:** 1 CNRS, Axe Environnement et Santé, Projet Ressources Naturelles, UMR 6134, SPE, Université de Corse, Corte, France; 2 Laboratoire Genesis Lab, Groupe Aupal, route du Cucuron, Lourmarin, France; University of Delaware, Lewes, Delaware, USA

**Keywords:** microbial diversity, indoor environment, bacteria, fungi, high-throughput sequencing

## Abstract

Indoor environments may impact human health significantly since microbial diversity made of various bacteria, fungi, and other microorganisms constitutes the habitat’s microbiome. These microorganisms originate from diverse sources, such as the environment, humans, and pets. Understanding such microbial diversity is crucial for assessing related health impacts. The aim of this study was to investigate bacterial and fungal microorganisms from different surfaces in a set of houses on Corsica Island (southeastern France). We conducted sampling and high-throughput sequencing of PCR-amplified microbial DNA from 40 habitats (eight different surfaces tested for each) located on diverse regions of the island. Following nucleic acid recovery, we performed PCR targeting V3–V4 regions of 16S rRNA for bacteria and ITS1 region for fungi. Bioinformatics processing allowed the identification of distinct microbial species and the analysis of their distribution. Our findings highlighted that the most represented bacterial genera in the Corsican homes tested were *Ralstonia* (16.9%), *Staphylococcus* (4.8%), *Corynebacterium* (2.7%), *Enhydrobacter* (6.8%), and *Methylorubrum* (6.0%). Regarding fungal diversity, the most common genera identified were *Dipodascaceae* (21.6%), *Rhodotorula* (10.9%), *Aspergillus* (8.9%), *Clavispora* (7.3%), and *Candida* (6.8%). Our results also showed differences in microbial composition among the habitats studied, while a higher bacterial and fungal diversity was observed in rural habitats and in those with pets.

**IMPORTANCE:**

This study provides valuable insights into the microbial diversity present in indoor environments of Corsican homes, specifically highlighting bacterial and fungal communities on various household surfaces. By identifying the predominant microbial genera and revealing differences linked to habitat characteristics, such as rural settings and pet ownership, this research enhances our understanding of how indoor microbial communities vary.

## INTRODUCTION

Whether at home, at work, or in other enclosed spaces, populations spend most of their days in an indoor environment, with a potential exposure to a broad range of microorganisms, including bacteria, fungi, and other microbial organisms. These microorganisms can be associated with various sources, such as surfaces, humans, pets, indoor plants, and ventilation systems, creating unique microbial signatures within each space (1).

Indoor microbial diversity is remarkably rich and complex. According to Rintala et al. (2), up to 1,000 different species can be present in household dust. Previous studies have revealed a great bacterial and fungal variety residing in these environments, forming what is known as the home microbiome (3–5). Research on indoor microbial diversity frequently highlights certain bacterial and fungal genera as predominant. For bacteria, genera such as *Staphylococcus*, *Corynebacterium*, *Acinetobacter*, and *Pseudomonas* linked to human skin, respiratory secretions, and dirty surfaces are among the most commonly detected (3, 6). Additionally, *Bacillus* and *Micrococcus* are widely distributed and commonly associated with dust and soil particles (7). When considering fungi, genera such as *Aspergillus*, *Penicillium*, *Cladosporium*, and *Alternaria* tend to dominate, as they adapt well to varying humidity levels and produce airborne spores, which are frequently found indoors (8).

The microbial diversity of indoor homes is influenced by various environmental and anthropogenic factors. Each habitat exhibits its own microbiome, influenced by factors such as the environment (1, 9, 10), humidity, ventilation, habitat type (7, 11, 12), human occupancy, and the presence of pets (8, 13, 14). Interestingly, investigations reveal that fungal communities within homes are primarily influenced by the outdoor environment, while bacterial communities are more likely to be modulated by occupants and pets (7, 11).

The indoor microbiome plays a dual role in human health. On one hand, microbial imbalances or overrepresentation of certain taxa can lead to health concerns, such as respiratory allergies or infections. Indeed, *Escherichia coli*, *Salmonella*, and *Legionella* can lead to gastrointestinal or respiratory infections (3, 15). Studies have shown that excessive exposure to biological agents, such as *Legionella pneumophila* and some *Penicillium* sp., in enclosed spaces can be linked to health problems (16). On the other hand, the presence of microorganisms in homes does not necessarily result in the occurrence of a disease. Ege et al. suggested that exposure to a diversity of microorganisms in our environment can be beneficial for strengthening our immune system and reducing the risk of developing allergies, particularly in the agricultural context (17). Douwes et al. demonstrated that exposure to a reduced richness of fungal and bacterial communities in household dust early in life can increase the risk of developing asthma later (16). In addition, some species of the genera, such as *Lactobacillus* and *Acinetobacter*, can protect against allergies (17).

The microbial communities within homes are known to be influenced by a variety of factors, yet our understanding of how these communities vary in unique and underexplored geographical regions, such as islands, remains limited. This knowledge gap is particularly relevant given the potential implications of indoor microbial diversity for human health and well-being. In this study, we wanted to explore the microbial diversity in indoor locations of the Mediterranean Corsica Island, focusing on bacteria and fungi. Specifically, we investigated how home characteristics, presence of human and non-human occupants, and geographical locations would influence the composition of microbial assemblages found in houses. To achieve this, we used high-throughput sequencing coupled with bioinformatics analyses in order to identify how home characteristics and the types of bacteria and fungi found outside home can shape the microbial assemblages collected from various surfaces inside homes.

## MATERIALS AND METHODS

### Sample collection

**Fig 1:**
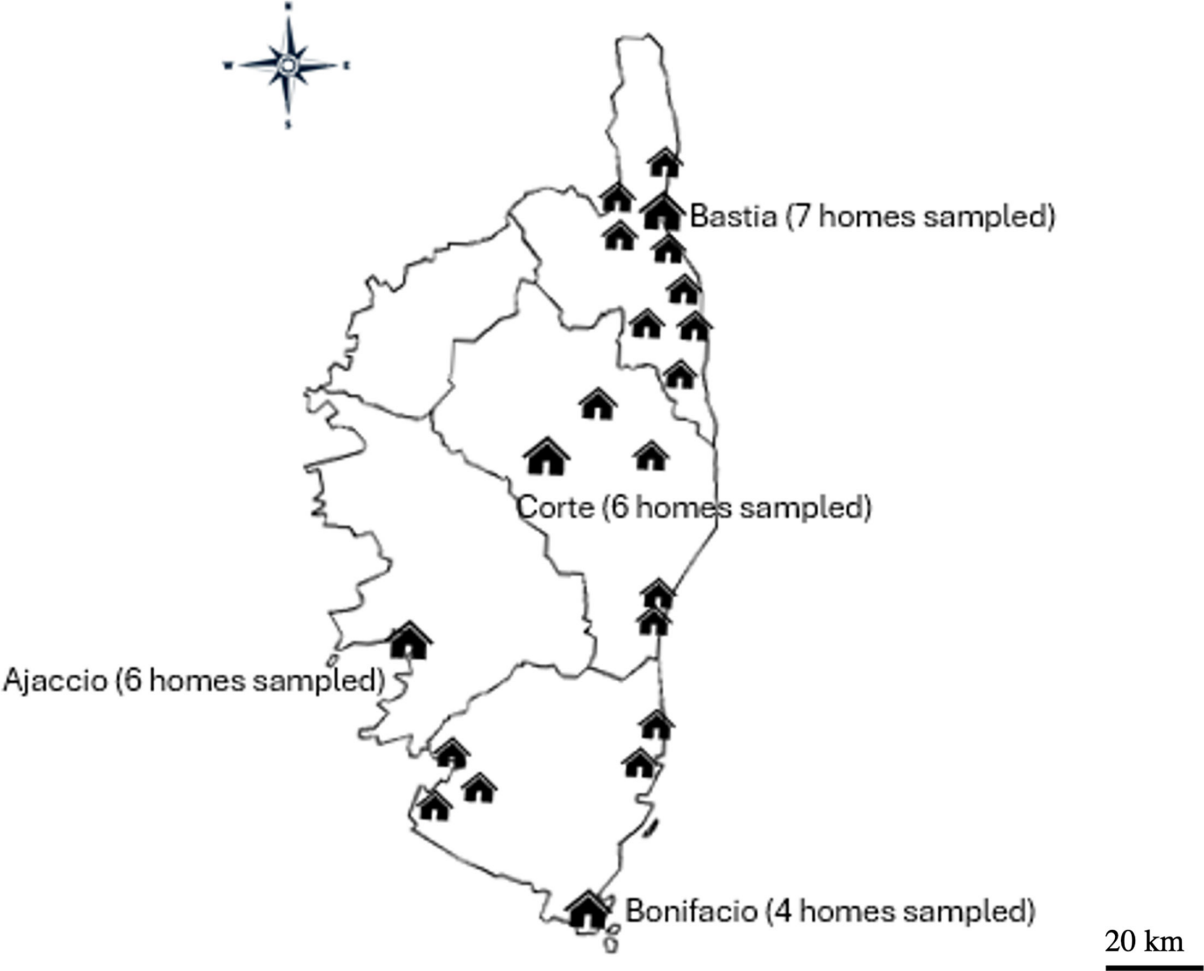
Geographic distribution of the 40 homes sampled.

To characterize the microbial diversity in the Corsican residential environments entering the study, we analyzed a total of 320 samples, in duplicate. Forty homes across the island of Corsica (Fig. 1) were selected for this study, with informed consent obtained from all participants.

A sampling kit was prepared for each participant, including a consent form and an anonymous questionnaire designed to collect extensive information about the household environment (summarized results of the questionnaire can be found in Table S1). This approach ensured a thorough and standardized collection of microbial samples across the diverse living spaces. Sampling was carried out by the participants of the study from May to June 2022 and focused on eight distinct surfaces, i.e., kitchen countertop, interior of refrigerator, TV screen, living room floor, front door handle, door frame, shower/bathtub floor, and toilet seat. Each swab was then cut using a sterile blade and placed in a sterile 1.5 mL microcentrifuge tube before being stored at −80°C until analysis.

### DNA extraction

DNA resuspension was achieved by adding 300 µL of phosphate-buffered saline (PBS) to the pre-cut swab tips. The various solubilized samples were then placed in a water bath at 60°C with agitation for 15 minutes. The extraction of nucleic acids originating from each sampling swab was performed using a KingFisher Flex apparatus and the MagMax viral/pathogen Ultra kit (both from ThermoFisher, Waltham, Illkirch, France) allowing extraction of nucleic acids from Gram-positive and Gram-negative bacteria, yeasts, and fungi. The test sample volume was 200 µL of the homogenized sample, with a final elution volume of 60 µL following the manufacturer’s protocol. A treatment of extracted DNA using the Rolling Circle Amplification (RCA) method was applied subsequently, in order to optimize the potential of characterization of the diverse microbial communities (18). For each sample, 10 µL of DNA extract was transferred to screw-cap tubes and denatured at 94°C for 3 minutes, followed by immediate cooling on ice. Then, 10 µL of RCA reaction mix (phi29 DNA polymerase, Thermo Fisher Scientific) was added to each tube, including a negative control, and the tubes were vortexed and briefly centrifuged. The reactions were incubated overnight at 30°C in a water bath. The following day, the reactions were inactivated at 65°C for 10 minutes. Recovered nucleic acids were stored at −25°C.

### PCR amplification

For the detection and identification of bacterial and fungal sequences, we performed a universal PCR targeting the V3–V4 regions of the 16S rRNA gene for bacteria and the ITS region for fungi. PCR amplifications for bacteria and fungi were carried out separately. Each reaction mixture consisted of sterile water, forward and reverse primers, and a Brillant Master Mix (Agilent Technologies), with 15 µL of mix added to 5 µL of DNA extract per well. PCR conditions were as follows: an initial denaturation at 95°C for 3 minutes; 25 cycles of 95°C for 30 seconds, 55°C for 30 seconds, and 72°C for 30 seconds; followed by a final extension at 72°C for 5 minutes and a hold at 20°C for 1 minute. According to Klindworth et al. (19), the V3–V4 and ITS hypervariable regions were chosen since they allow a fairly accurate identification of bacteria and fungi in a microbiome. For bacterial identifications, primers 16S_NGS_fwd (5′-TCG-TCG-GCA-GCG-TCA-GAT-GTG-TAT-AAG-AGA-CAG-CCT-ACG-GGN-GGC-WGC-AG-3′) and 16S_NGS_rev (5′-GTC-TCG-TGG-GCT-CGG-AGA-TGT-GTA-TAA-GAG-ACA-GGA-CTA-CHV-GGG-TAT-CTA-ATC-C-3′) were synthesized to harbor a 3′ portion specific to the 16S region to be amplified and a 5′ portion identifiable by the Illumina platform during subsequent high-throughput sequencing steps (19). For fungal identification, the Internal Transcribed Spacer (ITS), a non-coding and highly polymorphic region of ribosomal DNA, was amplified using the primers ITS_NGS_fwd (5′-TCG-TCG-GCA-GCG-TCA-GAT-GTG-TAT-AAG-AGA-CAG-CTT-GGT-CAT-TTA-GAG-GAA-GTA-A-3′) and ITS_NGS_rev (5′-GTC-TCG-TGG-GCT-CGG-AGA-TGT-GTA-TAA-GAG-ACA-GGC-TGC-GTT-CTT-CAT-CGA-TGC-3′) (20). The expected size of the amplicons was around 550 bp. Controls were included during each PCR step to ensure amplification specificity and detection of any potential contamination. Sterile water (H₂O) was used as a negative control to monitor for reagent or handling contamination. No amplification was detected in these negative controls. Amplification products were checked on agarose gels, purified using the MicroElute Gel Extraction Kit (Omega, Norcross, Georgia, USA) according to the manufacturer’s protocol, and resuspended in 20 µL of elution buffer.

A second PCR, with indexed primers (Illumina, San Diego, California, USA) specific for each amplified sample, was performed on purified amplicons from the first PCR. This indexing PCR was conducted in a final volume of 20 µL, using Illumina Nextera XT index primers and Brilliant Master Mix. PCR conditions included an initial denaturation at 95°C for 3 minutes, followed by 8 cycles of denaturation at 95°C for 30 seconds, annealing at 55°C for 30 seconds, and extension at 72°C for 30 seconds. A final elongation step was carried out at 72°C for 7 minutes, followed by a hold at 20°C for 1 minute. The expected size of amplicons was about 660 bp. Each amplification product was checked on an agarose gel and purified using the MicroElute Cycle-Pure Kit (Omega, Norcross, Georgia, USA). Amplicons were stored at −25°C. After quantifications using Qubit 4 Fluorometer (Thermo Fisher Scientific, Illkrich, France), the resulting libraries were analyzed by high-throughput sequencing on an iSeq100 platform (Illumina, San Diego, California, USA).

### Data analysis

Bioinformatic analyses (~50,000 to ~200,000 reads per sample) were performed using dedicated servers and software (Galaxy Pasteur, Galaxy Europe) (21). Taxonomic assignments were conducted using the Ribosomal Database Project (RDP) Classifier with the 16S (for bacteria) and ITS (for fungi) databases, using an assignment confidence cutoff of 0.5, the 'allrank' output format, and the 16S rRNA and fungal its_unite gene training model. Visualization of taxonomic profiles was carried out using KronaTools (22). Sequences were classified directly at the genus (bacteria) or order (fungi) level without prior OTU or ASV clustering, based on direct read assignment. Comparison with public databases (RDP reference database within Galaxy Pasteur) enabled the identification of microbial diversity patterns and the estimation of the relative abundance of detected taxa. Previous studies have shown that, in certain contexts, analyzing a high number of samples with moderate sequencing depth (100-1,000 reads per sample) can be sufficient to capture microbial community dynamics (23, 24). Thus, to ensure comparability and robustness, only genera/orders with at least 1,000 assigned reads were considered for downstream analyses. This approach was preferred because short-read amplicons from the targeted regions (V3–V4 for bacteria, ITS for fungi) do not always provide reliable species-level identification, and broader taxonomic ranks (genus/order) ensure more robust and comparable results across large-scale indoor microbiome studies. Alpha diversity was assessed using two standard metrics: richness (number of observed genera/orders) and the Shannon diversity index. Beta diversity was evaluated by calculating Bray–Curtis dissimilarities, followed by Non-Metric Multidimensional Scaling (NMDS) and Principal Coordinates Analysis (PCoA) for community visualization. Statistical comparisons were performed using Wilcoxon–Mann–Whitney tests or Kruskal–Wallis tests for alpha diversity, and PERMANOVA (adonis2 function) for beta diversity comparisons, with adjustments for multiple comparisons when appropriate. Both univariate models (analyzing each variable separately) and multivariate models (including several variables simultaneously, e.g., pets, geographic location) were tested. The multivariate approach yielded results consistent with the univariate analyses, suggesting that potential co-correlations between variables did not substantially influence the observed patterns.

## RESULTS AND DISCUSSION

### Global microbial diversity

In this study, we analyzed household dust samples collected from a variety of Corsican homes, which are selected to represent different domestic environments across the island. Corsica offers a unique setting for indoor microbiome studies due to its Mediterranean climate, marked seasonal variations, and diverse topography, which may shape indoor microbial communities in ways that differ from those observed in mainland regions.

We identified a total of 125 bacterial orders and 90 fungal orders, with almost all individual samples having at least 50 orders identified. Taxonomic identification showed that the dominant bacterial orders (in terms of sequences) represented 78.5% of the total relative abundance of bacteria in the entire 16S rRNA gene sequences analyzed. At the order level, the composition was strongly dominated by *Micrococcales*, *Burkholderiales*, and *Bacillales*. Previous studies have shown that the bacterial community in household dust is dominated by Gram-positive bacteria, particularly *Firmicutes* and *Actinobacteria* (2, 3, 23, 25). Orders such as *Burkholderiales* and *Bacillales* observed in our study align with previous research on indoor environments, since such orders have been frequently reported due to their association with human skin, dust, and moist surfaces (3, 6–8). Gram-negative bacteria were also found with mostly *Proteobacteria* (3).

**Fig 2:**
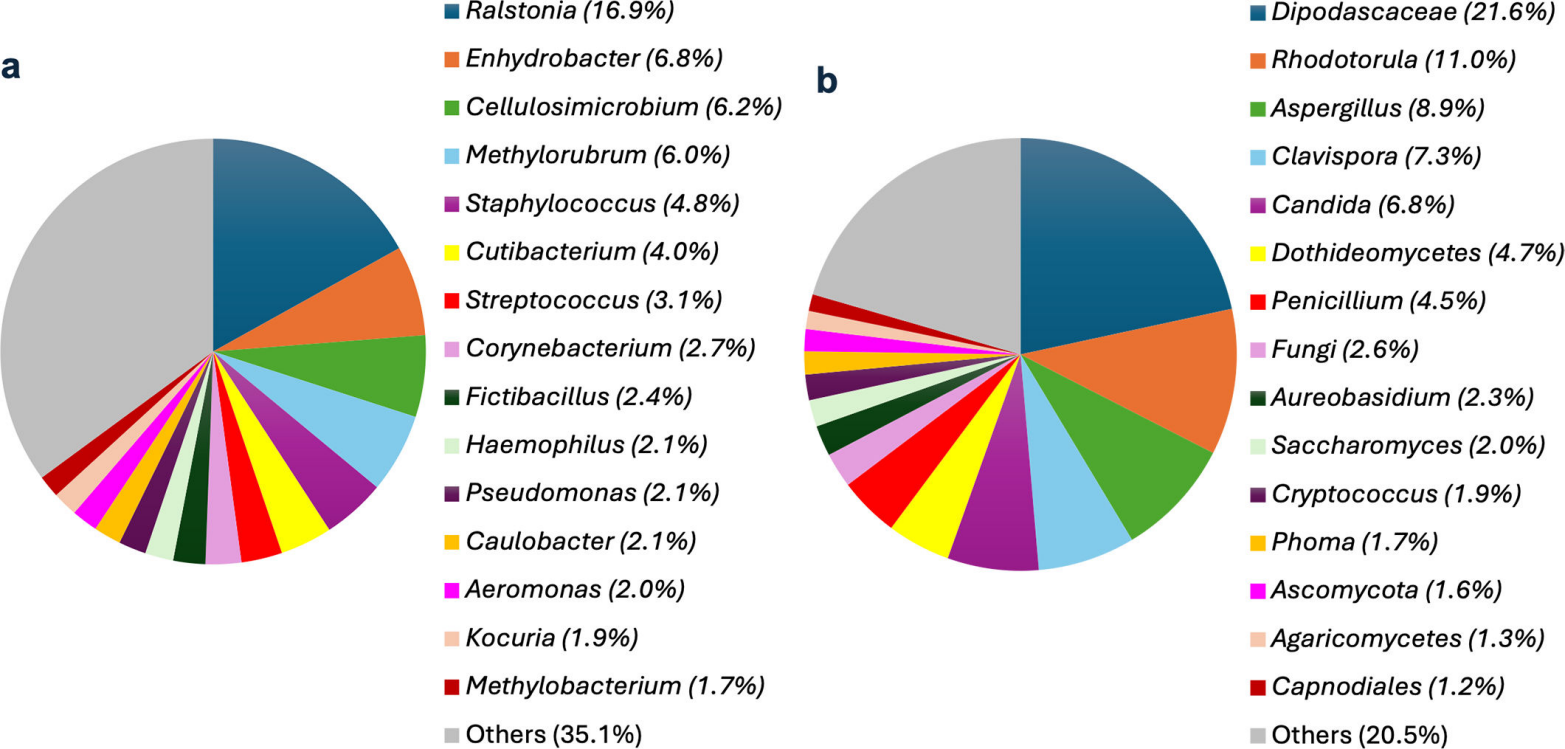
Relative abundance (%) of the 15 bacterial (**a**) and fungal (**b**) genera most abundant in habitats, with over 1,000 sequences.

The most abundant and represented bacterial genera within the habitats entering our study were *Ralstonia* (16.9%), *Enhydrobacte*r (6.8%), *Cellulosimicrobium* (6.2%), and *Methylorubrum* (6.0%) (Fig. 2a). Genera associated with human skin were also identified, including *Staphylococcus* (4.8%), *Streptococcus* (3.1%), and *Corynebacterium* (2.7%). According to Hanson et al. (3), the most common genera in household dust are *Bacillus*, *Enterococcus*, *Corynebacterium*, *Lactococcus*, *Staphylococcus*, *Micrococcus*, and *Streptomyces*. Our results are in agreement with this study for the genera *Staphylococcus* and *Corynebacterium*. The other genera were also identified in our study with relative percentages of less than 1%. In a study conducted in Belgian homes, the most common bacterial genera were *Staphylococcus*, *Paracoccus*, and *Sphingomonas* (9). In the United States, *Staphylococcus* and *Corynebacterium* were also dominant in household dust, while *Bacillus* and *Micrococcus* appeared at lower abundances (7). A study conducted in Singapore in 2012 in residential apartments showed that the most abundant bacterial genera were *Staphylococcus* and *Micrococcus* (26). In a Finnish study, the most represented genera in homes were *Streptococcus*, *Moraxella*, and *Staphylococcus* (27). Previous studies have emphasized how geographic location, climate, and household factors, such as the presence of pets, housing type, and lifestyle characteristics, likely shape the microbial communities, with *Ralstonia*, *Enhydrobacter*, and *Cellulosimicrobium* standing out as distinct to Corsican homes. These bacterial genera are often found in environmental sources such as soil and water, indicating that the unique geographic and climatic conditions of Corsica, along with local household characteristics, may favor their presence indoors.

Regarding fungi, the dominant orders represented 72.7% of the total relative abundance of fungi in the entire ITS rDNA gene sequences. The fungal composition was strongly dominated by *Saccharomycetales*, *Eurotiales*, and *Sporidiobolales*. Our results are in agreement with the literature, as *Saccharomycetales*, *Eurotiales*, and *Sporidiobolales* are frequently reported as dominant fungal orders in indoor environments (7, 28).

The most abundant fungal genera within the habitats were *Dipodascaceae* (21.6%), *Rhodotorula* (10.9%), *Aspergillus* (8.9%), *Clavispora* (7.3%), and *Candida* (6.8%) (Fig. 2b). Indeed, most identified fungi may originate from environments, such as soil (*Cryptococcus* and *Penicillium*), water (*Aureobasidium*), plants (*Cladosporium* and *Alternaria*), and human skin (*Candida*) (3, 23, 24). Previous studies have shown that the most abundant airborne fungi are *Alternaria*, *Penicillium*, *Aspergillus*, *Cladosporium*, *Aureobasidium*, and *Cryptococcus* (3, 29, 30). Our findings are consistent with studies conducted in Europe, where fungal genera such as *Aspergillus*, *Penicillium*, *Cladosporium*, and *Alternaria* have been commonly reported in indoor environments (31). The genera *Penicillium*, *Cladosporium*, and *Aspergillus* were found to be dominant in US residences, according to Adams et al. (32). Adams et al. (6) also have identified various fungal taxa, such as *Cladosporium*, *Aureobasidium*, *Penicillium*, *Phoma*, *Rhodotorula*, and *Alternaria*, which are known as plant pathogens, wood-decaying fungi, or yeasts. The genera *Cladosporium*, *Fusarium*, and *Curvularia* were the most prevalent in Mexican homes. In contrast, in Delhi, India, *Cladosporium*, *Alternaria*, and *Aspergillus* were the most represented (33). These results show how geographical location, climate, and domestic characteristics may influence fungal communities, with the dominance of *Dipodascaceae*, *Rhodotorula*, and *Clavispora* distinguishing Corsican homes from those in other regions. These fungal taxa are commonly associated with outdoor environments, such as plants and soil, suggesting that the Mediterranean climate and specific indoor–outdoor interactions in Corsican homes influence the fungal community composition.

In indoor environments, many bacterial and fungal genera coexist naturally, with some playing commensal or opportunistic roles. These microorganisms are generally harmless under normal conditions and, in certain cases, contribute to maintaining microbial balance and protecting human health. In the literature, certain bacterial genera, such as *Bacillus*, have been shown to produce enzymes that can degrade harmful fungal cell walls, thus contributing to a balanced microbial community (6, 10). Similarly, *Aspergillus* may compete with *Staphylococcus* for nutrients, and *Penicillium* is known to produce natural antibiotics that inhibit the growth of certain bacteria such as *Botrytis cinerea* and *Mucor miehei* (34). *Pseudomonas* species can also produce metabolites affecting the growth of fungi, such as *Candida* (28). While our study does not directly investigate microbial interactions, the simultaneous presence of these genera in our samples suggests the potential for such interactions as described in previous studies.

Interestingly, *Lactobacillus*, *Corynebacterium*, and *Neisseria* have been identified. They are known for their protective effects and are often considered commensal (35, 36). Indeed, Ege et al. (17) reported that *Lactobacillus*, *Neisseria*, and *Corynebacterium* identified in their study are inversely associated with asthma and allergies. However, under conditions of microbial imbalance, such as immune suppression, environmental stress, or changes in microbial diversity, commensal or opportunistic species may become pathogenic (37). Environmental stressors, such as increased humidity or temperature fluctuations, can promote the growth of pathogenic fungi, such as *Aspergillus* and *Candida* (37–39). Additionally, disruptions in microbial interactions, such as the overgrowth of one genus due to antibiotic use, can lead to pathogenic dominance (38).

We identified various genera considered to be pathogenic, such as *Aspergillus, Candida, Penicillium*, and *Cryptococcus*. Fungi such as *Aspergillus*, *Penicillium*, *Cladosporium*, *Acremonium*, *Paecilomyces*, and *Mucor* are associated with respiratory and allergic infections (40). The genera *Alternaria*, *Penicillium*, *Aspergillus*, and *Cladosporium* identified in our study are strongly associated with allergic respiratory diseases, particularly asthma (16). Moreover, according to Sharpe et al. (12), exposure to fungal species, such as *Aspergillus*, *Penicillium*, *Cladosporium*, *Ulocladium*, *Acremonium*, *Epicoccum*, *Scopulariopsis*, *Trichoderma*, *Alternaria*, and *Wallemia*, can increase respiratory symptoms in asthmatic patients living in homes with high fungal abundance.

Among the bacterial genera identified in our study, several contain species that are potentially pathogenic to humans and were present in all the habitats studied. For example, we identified the genus *Staphylococcus*, which is responsible for numerous human diseases and described as a risk taxon for childhood asthma. Among the species within this genus, certain strains of *Staphylococcus aureus* are particularly concerning due to their association with a broad spectrum of illnesses, including skin infections, pneumonia, and antibiotic-resistant infections (13, 41, 42). The presence of *S. aureus* in the corresponding samples was confirmed here using a targeted PCR approach with primers specific to the nuc gene, a species-specific marker for *S. aureu*s (43), followed by cloning and Sanger sequencing (not shown). This assay enabled species-level identification but did not provide information on strain type or antibiotic resistance profile. The *Lactococcus* and *Pseudomonas* genera, which are associated with asthma, and *Cutibacterium*, associated with eczema, were also identified (25, 38, 40, 44–46).

**Fig 3:**
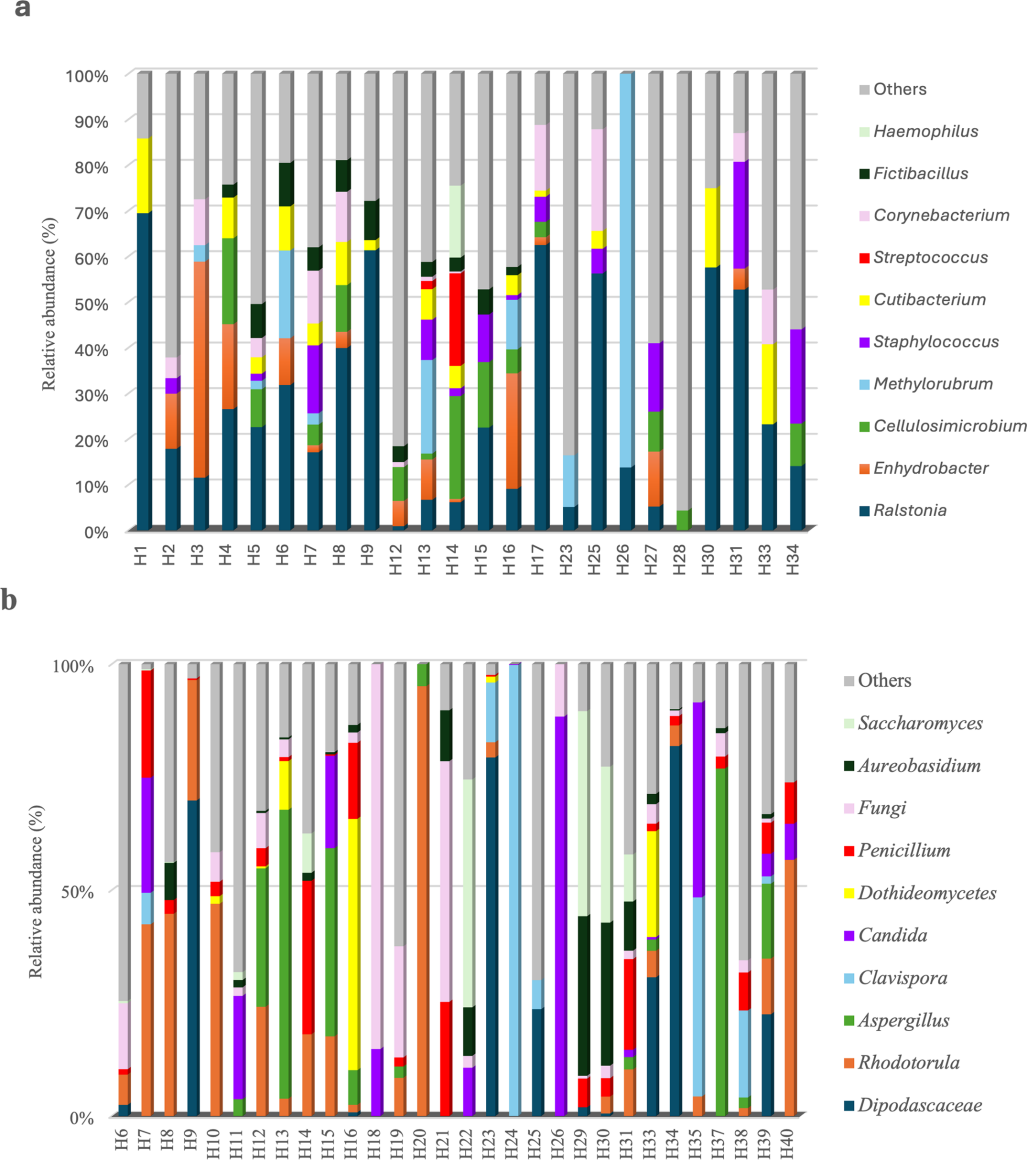
Relative abundance (%) of the 10 most abundant bacterial (**a**) and fungal (**b**) genera across habitats (H#).

Statistical analyses revealed that microbial community composition varied significantly across habitats, both for bacterial (PERMANOVA, *R*² = 0.335, *P* = 0.001) and fungal communities (PERMANOVA, *R*² = 0.545, *P* = 0.001). Certain genera, such as *Ralstonia* and *Cutibacterium*, were present in nearly all habitats tested, suggesting that they may form part of a shared domestic microbial community (Fig. 3a). However, their relative abundance varied significantly between habitats. For instance, *Ralstonia* dominated in several habitats (H1, H9, H17, H25, H30, and H31), whereas genera more closely associated with human activity, such as *Streptococcus* or *Staphylococcus*, showed increased prevalence in habitats H14 and in habitats H7, H31, and H34, respectively. These patterns may reflect differences in human activity levels or population density (23).

Figure 3b highlights similar marked variations among the 10 most abundant fungal genera. While *Rhodotorula* and *Penicillium* were present in nearly all habitats, their relative abundance varied considerably. For example, *Rhodotorula* was particularly dominant in habitats H20 and H40, whereas genera, such as *Aspergillus* and *Clavispora*, exhibited higher prevalence in H37 and H24, potentially due to environmental factors like humidity or the presence of specific building materials (6). The notable presence of *Candida* in certain habitats, such as H26, may reflect contributions from occupants through their skin or oral microbiomes, but *Candida* species are also known to originate from environmental sources, such as water, soil, and animals (47). These findings are in agreement with previous studies demonstrating that each household possesses a unique microbial signature influenced by a combination of environmental and anthropogenic factors. Local climate, the number and types of occupants, the presence of pets, and ventilation methods are all key contributors to this variation. A study on household dust in the United Kingdom and Greece identified a set of bacterial genera consistently present across the majority of homes in both regions. This shared bacterial community included *Acinetobacter*, *Massilia*, *Rubellimicrobium*, *Sphingomonas*, and *Staphylococcus*, which are commonly associated with human activity and environmental sources; however, significant regional differences were observed too. Thus, a higher abundance of *Rhizobiaceae* was detected in Greek homes. The authors attributed these variations to bioclimatic and regional factors, highlighting how geography and climatic conditions, such as those characteristic of the Mediterranean region, influence indoor microbial communities (48). However, in our study, *Rhizobiaceae* were indeed identified but were not among the most abundant bacterial families.

Occupants contribute significantly to the microbial diversity of their homes through their microbiomes and daily activities. Dunn et al. (20) showed that human skin, oral, and stool bacteria are key contributors to microbial communities on frequently touched surfaces, such as pillowcases, toilet seats, and door handles. Homes with larger families or children often harbor greater microbial diversity due to increased human activity and the mixing of individual microbiomes. Additionally, the presence of pets, particularly dogs, was found to increase diversity, as dog-associated bacteria, such as *Porphyromonadaceae* and *Pasteurellaceae*, were detected on surfaces like pillowcases and TV screens, even those without direct contact with the animals (23).

These observations reinforce the idea that the microbial communities of indoor environments are shaped by a dynamic interplay of factors, resulting in unique microbiome signatures for each habitat.

Accordingly, in addition to microbial distribution differences identified between habitats, we observed variations within the habitats between the different areas sampled.

### Microbial diversity according to sampled surfaces

**Fig 4:**
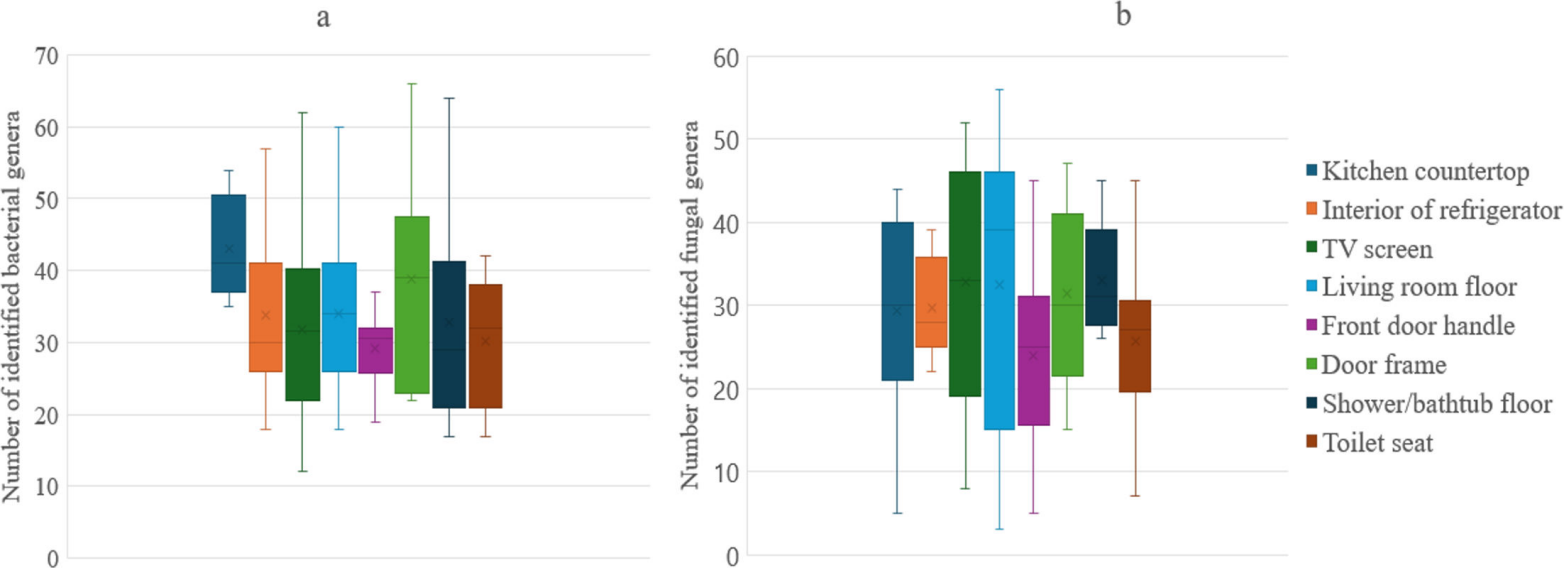
Average richness difference of bacterial (**a**) and fungal (**b**) communities between sampled surfaces.

Our results showed that microbial diversity varied considerably among the distinct surfaces tested, both for bacterial communities (PERMANOVA, *R*² = 12.9%, *P* = 0.001) and fungal communities (PERMANOVA, *R*² = 11.0%, *P* = 0.01). Surfaces such as the kitchen countertop (43 genera identified) and the door frame (38 orders identified), where aerosols and airborne particles are more likely to accumulate, tend to have higher and more variable diversity regarding bacteria (Fig. 4a). When considering fungi, the results varied more by surface, with the living room floor (42 genera identified) and the TV screen (37 genera identified) being particularly notable (Fig. 4b).

Conversely, some surfaces exhibited lower microbial diversity, defined here as having fewer than 30 identified genera. Regarding bacteria, the front door handle and toilet seat exhibited low diversity. For fungi, the door frame and toilet seat were among the least diverse surfaces, suggesting the conditions less conductive to the proliferation of microbial genera.

*Cellulosimicrobium*, *Ralstonia*, and *Caulobacter*, as well as fungal genera such as *Rhodotorula*, *Aureobasidium*, and *Aspergillus,* were found on all sampled surfaces. Analysis at the bacterial and fungal genus levels revealed variations in terms of the presence and/or proportion of bacterial communities depending on the surfaces tested.

**Fig 5:**
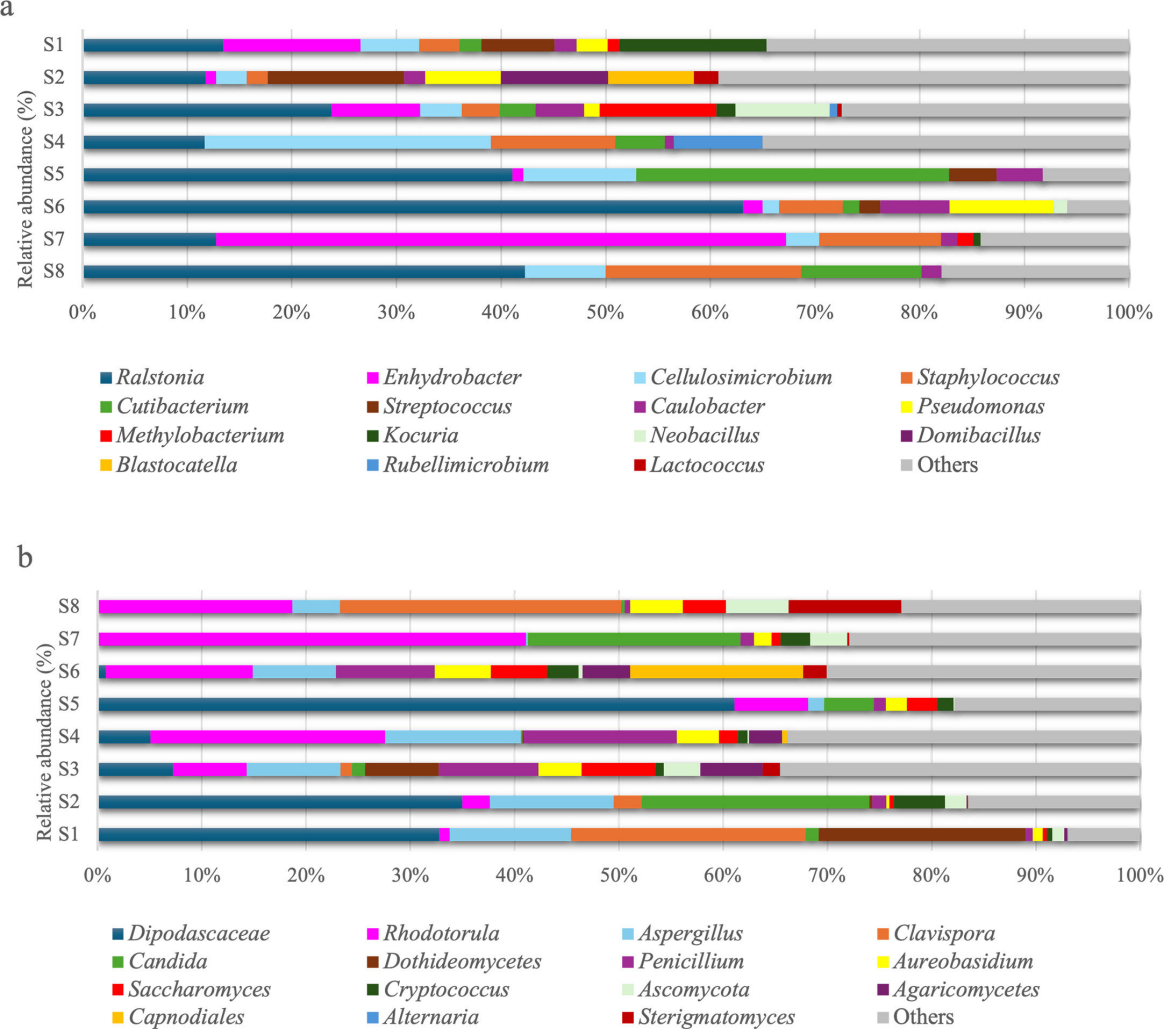
Relative abundance (%) of specific bacterial genera (**a**) and fungal genera (**b**) identified across sampled surfaces. Kitchen countertop (S1), refrigerator (S2), TV screen (S3), living room floor (S4), front door handle (S5), door frame (S6), shower/bathtub (S7), and toilet seat (S8).

For example, on the kitchen countertop, the bacterial genera *Enhydrobacter* and *Kocuria* are predominantly present, which were also found on other surfaces (Fig. 5a). When considering the refrigerator sampling, genera *Lactococcus*, *Pseudomonas*, *Blastocatella*, and *Domibacillus* were detected. Some of these bacteria are food-related, such as *Lactococcus* found in dairy and cheese production environments. Dunn et al. (23) demonstrated that *Pseudomonadales* taxa are relatively more abundant on kitchen surfaces. In our study, the genus *Pseudomonas* was also identified on kitchen surfaces (kitchen countertop and refrigerator), as well as on the TV screen and door frame. The fungal genera *Dipodascaceae*, *Clavispora*, and *Dothideomycetes* were the main genera found on the kitchen countertop, as well as genus *Aspergillus*, which was also present on the TV screen, door frame, and toilet seat (Fig. 5b). The genus *Candida* was mainly found in the refrigerator and on the shower and/or bathtub floor.

The bacterial genus *Rubellimicrobium* was only present on the living room floor and the TV screen. On the TV screen, the genus *Neobacillus* and *Methylobacterium* were specifically present; the same observation was made for the fungal genus *Penicillium*, which was present on the TV screen and other surfaces in smaller proportions. On the front door handle, the main bacterial genera were *Cutibacterium* and *Ralstonia*, while the fungal genus *Dipodascaceae* was the most represented. Also, the bacterial genus *Staphylococcus* was more abundant on the living room floor and the toilet seat. Indicator taxa analysis by Kembel et al. (49) highlighted bacterial communities associated with toilets, mainly belonging to taxa related to the intestinal or skin microbiota, including *Lactobacillus*, *Staphylococcus*, and *Streptococcus*. In our study, the genus *Streptococcus* was mainly present on kitchen surfaces (countertop and refrigerator). On the toilet seats, we mainly identified bacterial genera *Staphylococcus* and *Corynebacterium,* as well as *Ralstonia*, which is frequently found in water distribution systems (50). Genera *Winkia*, *Prevotella*, *Murdochiella*, *Lactobacillus*, and *Amaricoccus* were present exclusively on this surface. Most of these bacteria originate from the intestinal microbiota, as demonstrated in the study by Dunn et al. (23), showing that members of the *Clostridiales* and *Bacteroidales* orders represent generally the largest proportion of bacterial sequences collected from toilet seat surfaces. Regarding fungi, genera *Clavispora*, *Sterigmatomyces*, and *Divriesia* were retrieved in greater quantity on the toilet seat.

### Presence of pets (cats and dogs)

Literature data highlighted that variations in microbial communities within habitats are due to a series of variables such as the environment and habitat characteristics, as well as the presence or absence of animals and the lifestyle of the inhabitants. Thus, a comparison of bacterial and fungal diversity between habitats with and without animals was conducted in this study.

**Fig 6:**
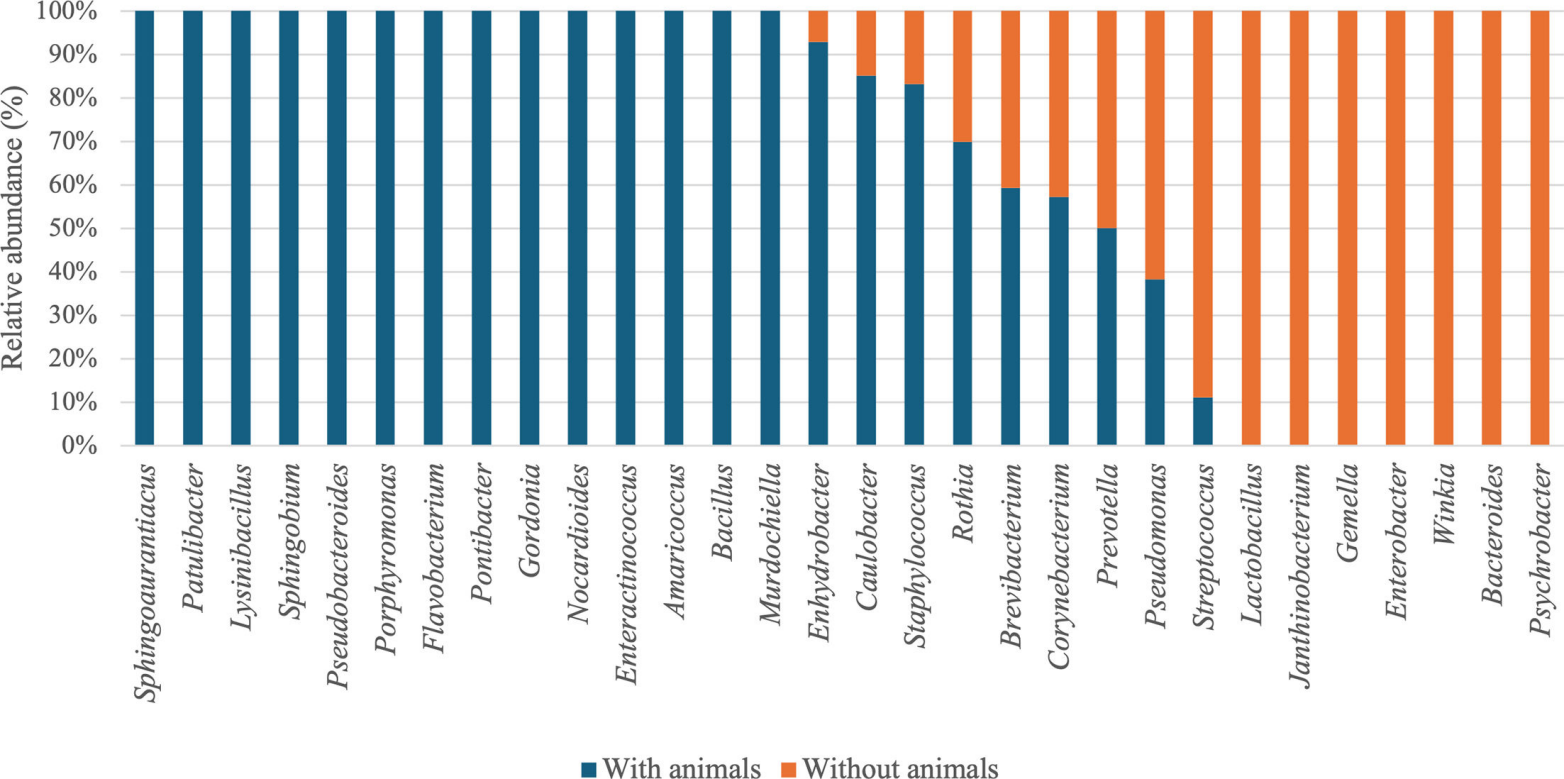
Average relative abundance (%) of bacterial genera based on animal presence.

Our results showed that habitats with animals exhibited significantly different microbial community compositions compared to habitats without animals, for both bacterial communities (PERMANOVA, *R*² = 2.2%, *P* = 0.035) and fungal communities (PERMANOVA, *R*² = 2.3%, *P* = 0.012). Habitats with animals exhibited significantly greater bacterial and fungal diversity, identifying 72 bacterial and 127 fungal genera, compared to 34 bacterial and 56 fungal genera in habitats without animals. These results are in line with literature data, as Kettleson et al. (8) found that homes with dogs contained four times more bacterial species than homes without dogs (8, 20). Fujimura et al. (13) also noted that bacterial diversity was higher in households with a dog. Numerous investigations have shown the impact of the presence of dogs on fungal and bacterial communities (8, 13, 14). Here, in habitats with animals, bacterial genera such as *Bacillus*, *Patulibacter*, and *Porphyromonas* were identified, while genera such as *Lactobacillus*, *Enterobacter*, and *Bacteroides* were retrieved in habitats without animals (Fig. 6).

Among the abundant genera found in habitats with animals, some, such as *Enhydrobacter*, *Staphylococcus*, *Flavobacterium*, *Norcardioides*, and *Porphyromonas*, were specifically associated with their presence. Several genera belonging to the *Proteobacteria* were associated with the presence of dogs. This is in accordance with the fact that this is the main phylum that inhabits the skin and intestines of dogs (14, 51). Barberán et al. (20) showed that bacterial diversity is higher in habitats with dogs and cats, with genera such as *Porphyromonas* present in the mouths and feces of dogs and cats. The authors suggested that the effect of pets on bacterial communities was partly due to their shedding of these bacterial taxa into homes. Dunn et al. (23) demonstrated that homes with animals exhibit a greater abundance of microbial taxa, such as *Porphyromonadaceae* and *Rhodocyclaceae*, which are abundant on the fur of dogs. Additionally, they observed that homes with animals had 42% more microbial richness compared to homes without animals (23). According to Hickman et al. (27), the presence of dogs is associated with the phylum *Proteobacteria*, particularly *Moraxellaceae* and *Rhodobacteraceae*, while the presence of cats is associated with *Micrococcus*. In our study, these bacterial families were identified in habitats with and without animals. Kettleson et al. (8) showed that *Nacordioides*, *Flavobacterium*, and *Staphylococcus* were more abundant in homes with dogs. Interestingly, certain genera identified in homes with animals, such as *Patulibacter*, *Sphingoaurantiacus*, *Lysinibacillus*, and *Bacillus*, are associated with the environment. Since some of these genera were found in soils outside habitats (52–55), these environmental bacteria may have been introduced into the habitats through the movements of pets during outdoor activities, as animals can act as vectors for microbial transfer by carrying environmental bacteria on their fur, paws, or through their feces. Conversely, dogs in the habitat would reduce the presence of human-associated genera, such as *Streptococcus* and *Prevotella* (56).

**Fig 7:**
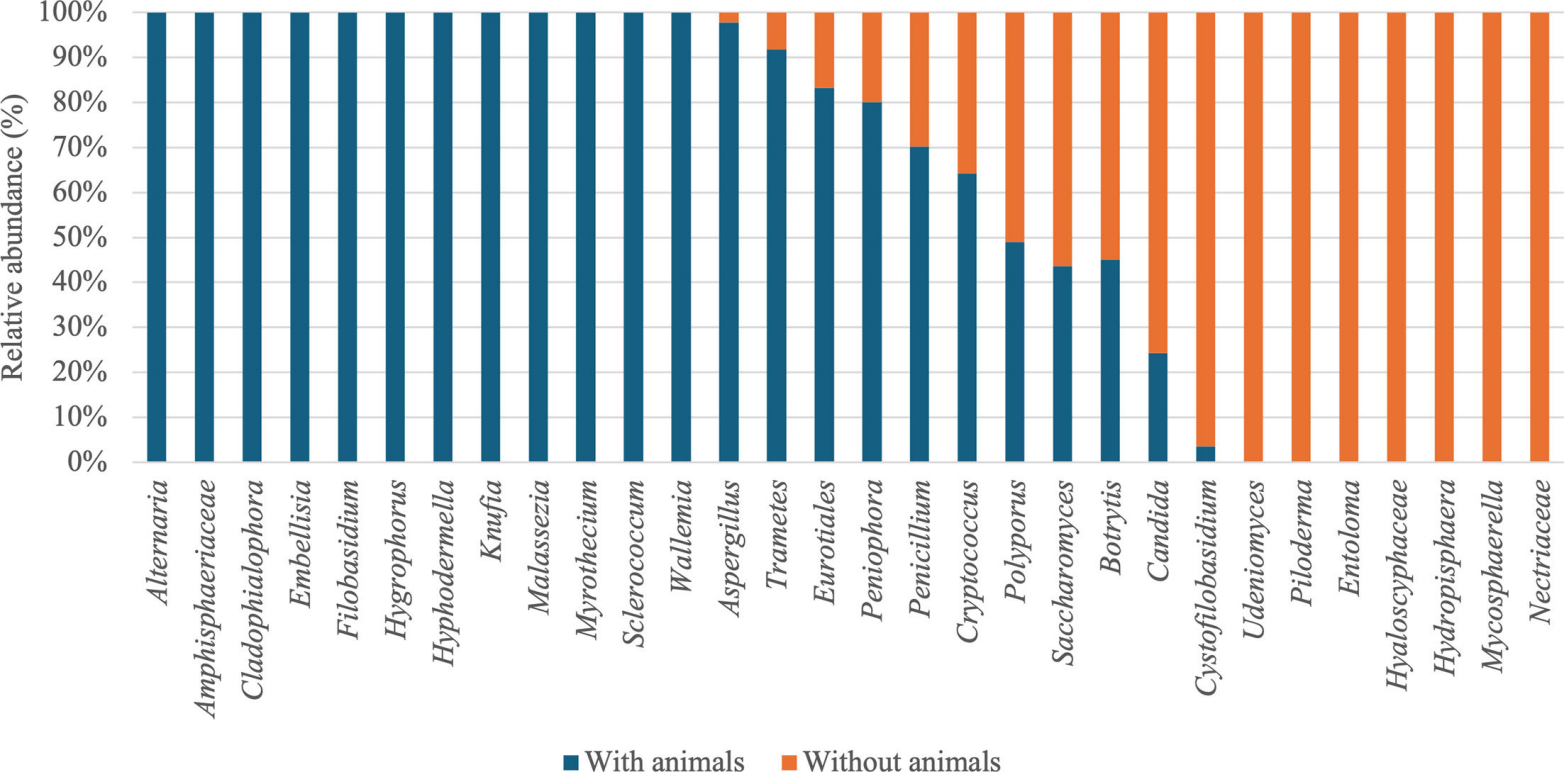
Average relative abundance (%) of fungal genera based on animal presence.

The same observation was made for fungi, with certain genera found exclusively in habitats with animals, such as *Alternaria*, *Amphisphaeriaceae*, *Cladophialophora*, *Embellisia*, *Filobasidium*, *Hygrophorus*, *Hyphodermella*, *Knufia*, *Myrothecium*, *Malassezia*, *Sclerococcum*, and *Wallemia*, while others were present in habitats without animals, such as *Piloderma*, *Udeniomyces*, *Entoloma*, *Hyaloscyphaceae*, *Hydropisphaera*, *Mycosphaerella*, and *Nectriaceae* (Fig. 7). The fungal genera found in habitats with animals and without animals are environmental genera. Indeed, some genera, such as *Alternaria*, *Amphisphaeriaceae*, *Entoloma*, *Filobasidium*, *Nectriaceae*, *Knufia*, and *Myrothecium*, are found in soil, water, and plants (57–65). However, a higher fungal diversity was observed in habitats with animals compared to those without. This higher diversity can be explained by the contribution of dogs and cats during their outdoor activities. Conversely, we did not observe significant differences on microbial diversity when considering inhabitants wearing shoes indoors or not (not shown).

In a study by Gómez et al. (66)*,* fungal species such as *Alternaria alternata*, *Mucor plumbeus, Cladosporium cladosporioides*, and *Malassezia restricta* were more abundant in habitats with animals. A study conducted by Hickmann et al. (27) showed that the presence of animals was responsible for variations in the relative abundance of certain fungal genera. The authors observed that the presence of dogs in a household is associated with a greater abundance of *Ascomycota* and, conversely, a lower abundance of *Basidiomycota*, consistent with our results.

We also compared bacterial diversity between habitats with dogs and those with cats. Twenty-two and twenty-eight bacterial genera retrieved only in homes with dogs and cats, respectively, were identified. Regarding fungal diversity, 29 and 107 genera were present only in homes with dogs and cats, respectively. We observed that the presence of dogs influenced both bacterial and fungal diversity, whereas the presence of cats had a more significant influence on fungal communities. This may be explained by the fact that most single-cat households are located in rural areas, where cats frequently move between indoor and outdoor environments. This increased exposure to the outside world likely facilitates the introduction of fungal spores from soil, vegetation, and other natural reservoirs into the home, thereby increasing fungal diversity. Diverse studies have highlighted the association between the presence of dogs and the composition of bacterial and fungal communities, with dogs having a lesser impact on fungal communities, which is consistent with our results (8, 20, 23). However, our results showed that the presence of cats was also associated with fungal diversity.

### Type of housing and geographic location

**Fig 8:**
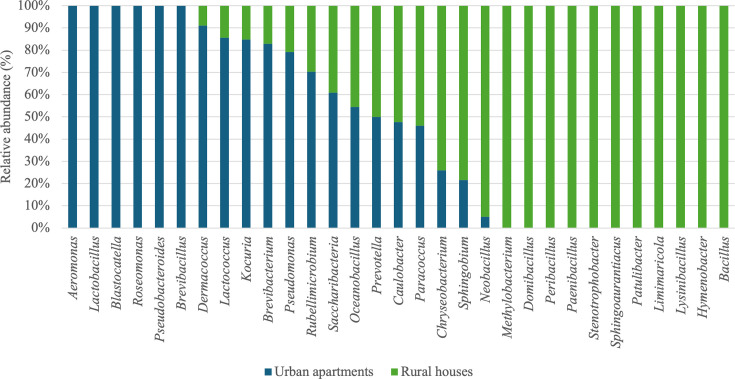
Average relative abundance (%) of bacterial genera based on type of housing and geographic location.

To estimate whether the type and location of housing in the Corsican area tested have an impact on the microbial communities identified, a comparison was made between types of habitats (urban apartments vs. rural houses). Statistical analyses showed that bacterial communities varied significantly according to geographic location (PERMANOVA, *R*² = 3.4%, *P* = 0.001), while fungal communities showed only a trend toward separation (PERMANOVA, *R*² = 1.9%, *P* = 0.073). In our study, all apartments were located in urban areas, while houses were located in rural areas. Homes in rural areas exhibited slightly higher bacterial diversity (64 genera identified) compared to urban areas (47 genera). A similar pattern was observed for fungal diversity, with houses in rural areas presenting a much higher fungal richness (around 120 genera identified) compared to apartments in urban areas (around 60 genera), although this difference was not statistically significant according to PERMANOVA analysis (*P* = 0.073). A study conducted by Nasir and Colbeck (67) revealed that a higher concentration of bacterial aerosols was found in houses than in apartments, due to a generally higher number of occupants in houses, highlighting more extensive sources and ecological niches than in apartments. Our analysis showed that certain bacterial and fungal genera are present only in houses, while others are only found in apartments. Thus, we observed that houses in rural areas exhibited greater bacterial diversity, with 18 additional bacterial genera identified. Among these, some are represented in Fig. 8, such as *Methylobacterium*, *Domibacillus*, *Peribacillus*, *Paenibacillus*, *Stenotrophobacter*, *Sphingoaurantiacus*, *Patulibacter*, *Limimaricola*, *Lysinibacillus*, *Hymenobacter*, and *Bacillus*.

The genus *Methylobacterium* was associated with the presence of vegetation and was more abundant in rural houses, as reported by Rai et al. (68). Most of the genera found exclusively in rural areas are linked to their environment, such as soils or plants (69–71). For example, *Paenibacillus* has been isolated from plant roots, while *Stenotrophobacter* and *Lysinibacillus* were derived from soils (72–74). To our knowledge, other genera identified have not yet been explicitly associated with rural houses in the literature. In contrast, genera such as *Pseudomonas*, *Aeromonas*, *Kocuria*, *Lactobacillus*, *Blastocatella*, *Rubellimicrobium*, *Dermacoccus*, *Lactococcus*, *Roseomonas*, and *Pseudobacteroides* are more abundant in urban areas, with some being present in these environments. These genera often reflect human activity, as supported by Hickmann et al. (23), who observed that Finnish urban environments are dominated by bacterial genera, such as *Pseudomonas*, which are linked to human activity and environmental pollution. Additionally, urban apartments are particularly enriched in genera associated with human occupations, such as *Lactococcus* and *Lactobacillus* (68). These findings emphasize the role of geographic location and human presence in shaping microbial communities, with rural areas being more associated with soil and vegetation-related bacteria, such as *Bacillus* and *Mycobacterium*, whereas urban areas are influenced by human-related and pollution-associated microbes.

**Fig 9:**
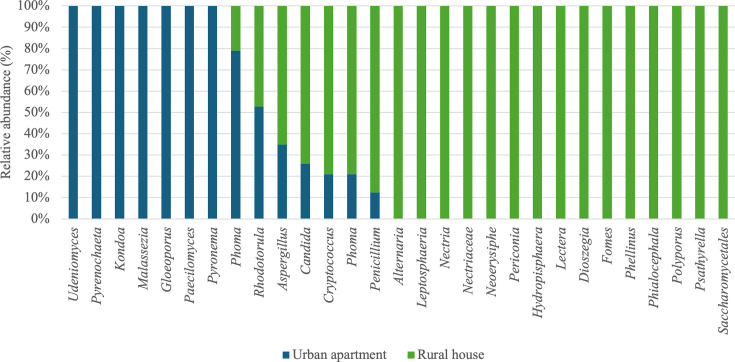
Average relative abundance (%) of fungal genera based on type of housing and geographic location.

Regarding fungal communities, genera such as *Alternaria*, *Dioszegia*, *Fomes*, *Hydropisphaera*, *Lectera*, *Leptosphaeria*, *Nectroa*, *Nectriaceae*, *Neoerysiphe*, *Periconia*, *Phellinus*, *Phialocephala*, *Polyporus*, *Psathyrella*, *Saccharomycetales*, and *Tumularia* were present in habitats located on rural areas only (Fig. 9). These findings are in accordance with previous studies associating such genera with environmental conditions typical of rural settings. For instance, *Aspergillus* and *Alternaria,* linked to decaying plant material, agricultural residues, and soils, were found in greater abundance in the rural areas included in our study (6, 75, 76).

A study by Hickmann et al. (27), comparing fungal communities based on habitat type, showed that genera such as *Aspergillus*, *Pseudopithomyces*, *Epicoccum*, and *Cladosporium* were more abundant in houses than in apartments, while the genera *Naganishia* and *Heterobasidion* were more abundant in apartments. Among these genera, only the genus *Aspergillus* was identified in our study. Certain genera, such as *Aspergillus*, *Candida*, *Cryptococcus*, *Penicillium*, and *Phoma*, were found in greater abundance in rural habitats. Barberan et al. (20) showed that geographic variability impacts the relative abundances of potential fungal allergens, such as *Alternaria*, *Aspergillus*, and *Phoma*, which are present in greater quantities in habitats in countryside, including the Great Plains, the Great Lakes, the Northeast United States, and Arizona. Our results are similar to this study, since the genera *Alternaria* and *Aspergillus* were found to be more abundant in rural houses than in urban apartments.

Finally, some studies have shown that bacterial communities are often less influenced by geographic location than fungal communities, reflecting differences in how these microorganisms respond to environmental factors. For instance, Barberán et al. (20) demonstrated that fungal taxa in indoor environments showed distinct biogeographic patterns tied to outdoor environmental conditions such as climate and vegetation, whereas bacterial taxa were more strongly influenced by human occupancy and indoor activities. This suggests that fungal communities are more dependent on external inputs like spores from surrounding environments, which vary significantly between rural and urban areas. Adams et al. (6) found that bacterial communities in the same environments were more homogeneously distributed due to their reliance on indoor human activities and built environment characteristics. In contrast, homes in rural areas or near natural landscapes exhibit higher fungal diversity, likely influenced by the surrounding vegetation. Thus, Dockx et al. (9) demonstrated that the presence of green spaces near habitats determined partially indoor microbial communities, with the proximity of such environments (within 100 m) increasing the abundance of fungal genera.

Other household parameters, such as the number of inhabitants, the presence of children, and the use of shoes indoors, were also evaluated but did not show statistically significant differences in microbial community composition (*P* > 0.05 for all comparisons). Although some comparisons (e.g., geographical location) yielded statistically significant results, the low *R*^2^ values indicate that these factors explain only a small proportion of the observed variation. This suggests that other unmeasured parameters may play a more substantial role in shaping the microbial communities.

### Conclusion

This study highlights the intricate balance within microbial communities in indoor environments. Our results showed that the most represented bacterial genera in these habitats were *Ralstonia*, *Enhydrobacter*, *Cellulosimicrobium*, *Methylorubrum*, and *Staphylococcus*. This study revealed significant differences in bacterial and fungal communities across the diverse Corsican habitats tested as well. Notably, homes with pets displayed a higher microbial diversity, particularly affecting fungal populations, confirming a significant impact of domestic animals on indoor microbial ecology. Homes with animals were characterized by the presence of bacterial genera such as *Bacillus*, *Patulibacter*, and *Porphyromonas*, while habitats without animals harbored genera like *Lactobacillus*, *Enterobacter*, and *Bacteroides*. In rural areas, the diversity of fungi was much greater, with a higher number of genera identified compared to urban habitats. This likely reflected the proximity of rural homes to natural environments and their exposure to a wider range of outdoor microbes. Furthermore, geographical location also influenced microbial composition, with genera such as *Methylobacterium*, *Domibacillus*, and *Peribacillus* being identified in specific areas, highlighting the role of local environmental factors in shaping indoor microbial communities.

These findings showed the complex dynamics of indoor microbial communities and underscore the influence of pets and rural environments on microbial diversity. Understanding these dynamics is crucial for assessing potential health risks and guiding future research on indoor microbiomes. While this study provides valuable insights into the microbial communities in Corsican homes, further research could explore the seasonal dynamics of microbial communities and examine their functional traits to gain a deeper understanding of their ecological roles. Expanding our approaches to different geographical regions and housing types would help to further uncover the broader patterns and drivers of microbial diversity in indoor environments.

## SUPPLEMENTAL MATERIAL

Table S1 aem.00756-25-s0001.docx Information from the 40 Corsican homes sampled.

ASM does not own the copyrights to Supplemental Material that may be linked to, or accessed through, an article. The authors have granted ASM a non-exclusive, world-wide license to publish the Supplemental Material files. Please contact the corresponding author directly for reuse.
