# Supplementary figures and images for: Characterization of the bacterial and fungal diversity in habitats of Corsica Island
Source: Appl Environ Microbiol. 2025 Oct 1;91(10):e00756-25. doi: 10.1128/aem.00756-25 (PMC12542654; doi:10.1128/aem.00756-25)

**Table S1: Information related to habitats entering the study**


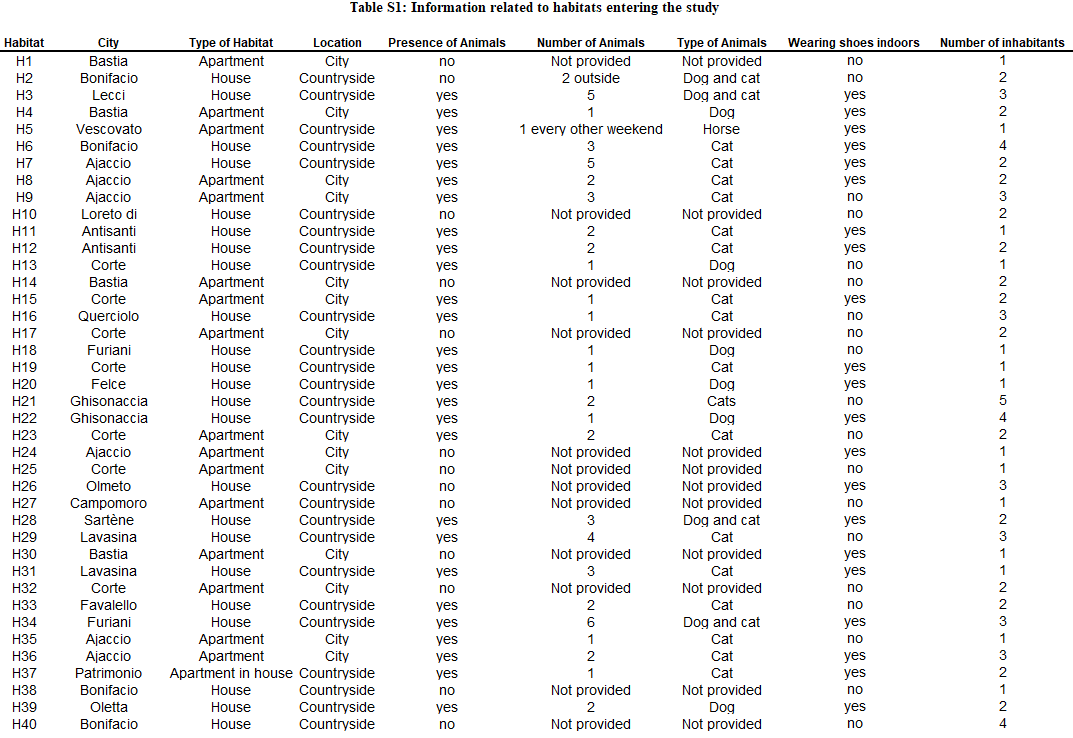

Supplement: Table S1 — Information from the 40 Corsican homes sampled. [file aem.00756-25-s0001.docx]
